# Interspecies Exchange of Mobile Genetic Elements During a Plant Disease Outbreak

**DOI:** 10.1093/gbe/evag192

**Published:** 2026-07-31

**Authors:** Daria Evseeva, Yann Pecrix, Marek Kucka, Catrin Weiler, Carolin Franzl, Markéta Vlková-Žlebková, Elena Colombi, Yingguang Frank Chan, Stéphane Poussier, Emmanuel Wicker, Honour C McCann

**Affiliations:** Research Group for Plant Pathogen Evolution, Max Planck Institute for Biology, Tübingen, Germany; CIRAD UMR PVBMT, Saint-Pierre, La Réunion F-97410, France; Friedrich Miescher Laboratory of the Max Planck Society, Tübingen, Germany; Research Group for Plant Pathogen Evolution, Max Planck Institute for Biology, Tübingen, Germany; Research Group for Plant Pathogen Evolution, Max Planck Institute for Biology, Tübingen, Germany; Research Group for Plant Pathogen Evolution, Max Planck Institute for Biology, Tübingen, Germany; Macquarie University, Sydney, Australia; Friedrich Miescher Laboratory of the Max Planck Society, Tübingen, Germany; CIRAD UMR PVBMT, Saint-Pierre, La Réunion F-97410, France; Université de la Réunion, La Réunion, France; CIRAD UMR-PHIM, Montpellier, France; CIRAD UMR-PHIM, Montpellier, France; Research Group for Plant Pathogen Evolution, Max Planck Institute for Biology, Tübingen, Germany

**Keywords:** microbial evolution, disease emergence, bacterial plant pathogen, mobile elements, integrative and conjugative elements, genomic epidemiology

## Abstract

Outbreak sequencing provides insight into the origin and evolutionary processes acting on emerging pathogens. Sequencing a historic multihost outbreak of *Ralstonia* spp. in Martinique shows the outbreak was caused by two lineages that diverged at separate times from mainland populations. One lineage (*Ralstonia pseudosolanacearum* I-18) was originally introduced from Asia to South America, where it became well established prior to its dissemination to Martinique, where it retains a signature of specialization on solanaceous hosts. The novel lineage first identified during the outbreak (*Ralstonia solanacearum* IIB-4NPB) arose from a mainland population endemic to the Americas prior to its arrival in Martinique, where host-range expansion was observed. In contrast to minor changes in secreted effector protein repertoires, the emergent *R. solanacearum* IIB-4NPB acquired a novel integrative and conjugative element (ICE^RsoRUN1145^). After identifying all *Ralstonia* spp. ICEs and mapping their spatial and phylogenetic distribution among *Ralstonia* spp. sampled during the outbreak, we found closely related ICEs circulating in mainland populations of *R. pseudosolanacearum*, indicating likely exchange between introduced and endemic *Ralstonia* spp. The family of ICEs in *Ralstonia* (ICERs) has a conserved bipartite structure and display a striking pattern of functional specialization in each cargo gene insertion hotspot: the first hotspot is a target for metabolic gene acquisition, and the second is a target for defense element acquisition. This work provides unparalleled phylogenetic and spatial resolution of an unusual outbreak and highlights the role of horizontal transfer in shaping the ecological success of an emerging pathogen.

Significance Dense outbreak sequencing in human populations has provided new insight into the processes underlying the emergence of novel pathogen lineages, yet far less is known about plant disease emergence. We sequenced a historic outbreak that swept across the island of Martinique, affecting an unusually wide range of hosts. The outbreak was caused by two different lineages that arrived on the island at different times but were often sampled from the same environment or host species. These co-occurring lineages sometimes share very similar mobile elements called integrative and conjugative elements (ICEs). *Ralstonia* ICEs specialize in the carriage of genes that increase opportunities for substrate utilization and confer resistance to viral predation, contributing to successful pathogen establishment.

## Introduction

Disease outbreaks on crop plants have severe consequences for subsistence and commercial growers alike, yet we have little understanding of the mechanisms underlying pathogen emergence in agricultural environments. Whole-genome sequencing of bacterial disease outbreaks has revealed the origins and evolutionary processes acting on emerging pathogens, highlighting particular role of mobile elements in resistance and virulence evolution (Azarian et al. 2018; Keller et al. 2019; Lassalle et al. 2023; Adams et al. 2025). Despite recent notable exceptions, most work in this area focuses on human pathogens and it is unclear how generalizable this work is to plant pathogens (McCann et al. 2017; Landa et al. 2020; Quibod et al. 2020; Colombi et al. 2024; Roman-Reyna et al. 2024; Timilsina et al. 2025). Plant disease can spread quickly across vast areas used for food, forage, and industrial crops, with severe consequences for agricultural productivity and economic stability. Clarifying the mechanisms underlying plant disease emergence is of both practical value and fundamental importance, deepening our understanding of how microbes adapt to new hosts and environments.

Members of the *Ralstonia solanacearum* species complex (RSSC) cause lethal infections in many different host species (Wicker et al. 2012; Genin and Denny 2012; Clarke et al. 2015). The species complex is comprised of *R. solanacearum* (Rso, also known as phylotype II and further subdivided into Rso IIA and IIB), *Ralstonia pseudosolanacearum* (Rps, phylotypes I and III), and *Ralstonia syzygii* (Rsy, phylotype IV) (Prior et al. 2016; Safni et al. 2018). Strains are typically assigned to one of over 56 sequevars using single or multilocus sequence analysis (MLSA) (Cellier et al. 2025). Since the pathogen is rapidly disseminated and can persist for years in freshwater and soil, extensive monitoring and movement controls are used to limit its spread. Clonally propagated crops like potato and banana are particularly vulnerable since pathogens can spread rapidly in areas with high host density and genetic homogeneity (McCann 2020; Drenth and Kema 2021; Dewberry et al. 2024). Multiple *Ralstonia* species and lineages are known to infect banana, and the threat posed to potato production is so great that one lineage (Rso IIB-1) is classified as a Select Agent by the United States Department of Agriculture (USDA 2026).

In April 1999, an unusual outbreak of disease caused by *Ralstonia* sp. was recorded in the Caribbean island of Martinique (Wicker et al. 2007). The host (*Anthurium* sp.) was not previously susceptible to *Ralstonia* sp. in Martinique and reports of additional infections in unusual hosts (*Cucumis*, *Cucurbita*, and *Citrullus* spp.) soon followed. The infections were caused by a novel lineage of *R. solanacearum* very closely related to Rso IIB-4, which is responsible for severe outbreaks in banana plantations across Central and South America, where banana is cultivated on an industrial scale (Wicker et al. 2007). At that time, banana plantations occupied 51% of cropland in Martinique (Food and Agriculture Organization of the United Nations 2001). Although initial inoculation trials indicated the new lineage was nonpathogenic on banana (hence Rso IIB-4NPB), the detection of field infections in banana and unusual host-range expansion event prompted an extensive survey for *Ralstonia* spp. across Martinique and the coastal mainland of French Guiana (Wicker et al. 2009; Deberdt et al. 2014). Although MLSA showed both *R. pseudosolanacearum* and *R. solanacearum* lineages were recovered during sampling, their origin, diversity, and genomic changes associated with the outbreak were unclear.

We sequenced 409 *Ralstonia* sp. isolated from between 1999 and 2014 from infected crops (81.7%), soil (14.1%), and freshwater (4.2%) environments during a disease emergence event characterized by the rapid and unprecedented expansion of Rso IIB-4NPB across a broad range of hosts (Wicker et al. 2007; Deberdt et al. 2014). We combined these data with 407 publicly available genomes to identify the origin and timing of diversification events leading to the outbreak, the spatial pattern of pathogen dissemination, and the genomic features associated with the ecological success of the host range–expanded lineage Rso IIB-4NPB.

We show here that the two dominant lineages sampled during the outbreak were both introduced into Martinique from well-established populations on the South American mainland. Rso IIB-4NPB, which caused natural infections in the most diverse group of hosts ever documented for a single lineage, is derived from an ancestral population endemic to the Americas. In contrast, Rps I-18, which displays a clear signature of host specialization, is derived from a population originally introduced into the Americas from Asia. Interestingly, horizontal exchange between Rps and Rso may have been responsible for the acquisition of an integrative and conjugative element (ICE) by Rso IIB-4NPB. The ICE acquisition is among the most significant genomic changes in the ancestor of the novel lineage prior to its dissemination and expansion across Martinique.

ICEs are large, self-transmissible mobile elements capable of both vertical and horizontal transmission (Botelho and Schulenburg 2021; Gomberg and Grossman 2024). An ICE will remain in the bacterial host chromosome until excision is triggered and it is transferred to a recipient cell by means of an ICE-encoded type 4 pilus (Delavat et al. 2017). In addition to the genes encoding core functions (excision, conjugation, and integration), ICEs carry accessory genes in hotspots of cargo gene integration. These are highly important vectors for the acquisition of genes encoding novel functions relevant to pathogenesis and resistance (Benigno et al. 2023; Colombi et al. 2024; Ryan et al. 2024). The ICE acquired by Rso IIB-4NPB carries clusters of metabolism-associated and bacterial defense system genes in two distinct hotspots of cargo gene integration, a pattern of functional specialization consistent across the entire family of ICEs in *Ralstonia* (ICERs) identified here. We also find an amplification of CRISPR-mediated phage defenses attesting to both a surge in viral encounter and enhanced resistance in the ancestor of the emergent lineage. In contrast, we found very few changes in the repertoire of secreted virulence proteins typically linked with pathogenesis. These results provide an important counterpoint to the view that plant pathogen emergence is solely driven by virulence factor acquisition or innovation, highlighting instead the importance of novel metabolic pathways and antiviral resistance for the ecological success of soil-borne pathogens.

## Results

### Global Phylogeography of the RSSC

**Fig. 1.**
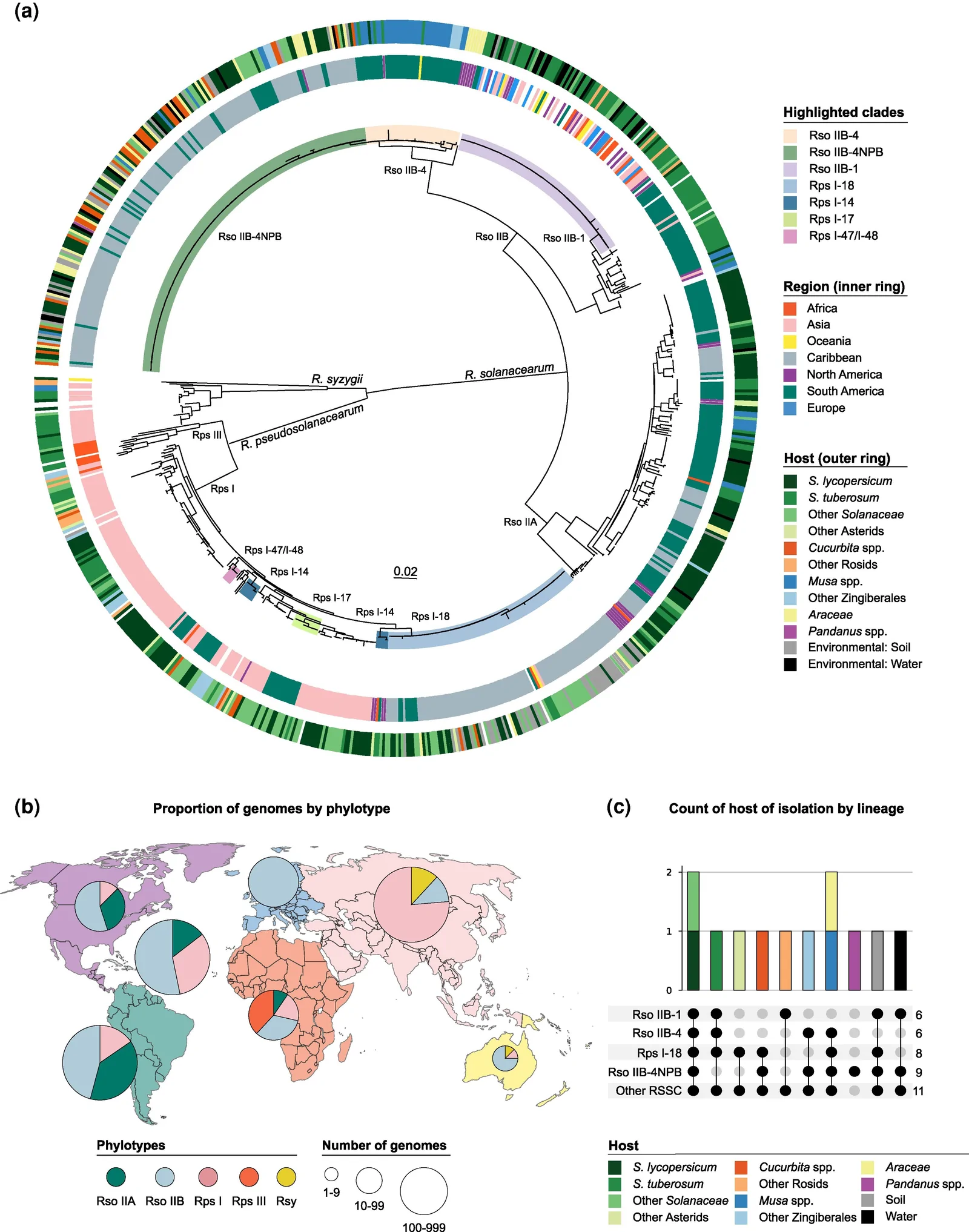
Global diversity and distribution of the *R. solanacearum* species complex. a) Maximum likelihood tree of 816 RSSC genomes inferred from 137,423 bp SNP alignment. Nodes with bootstrap support values under 70 are collapsed. Clades of interest are highlighted on the tree. Geographic region and host of isolation are shown in the inner and outer rings, respectively. Martinique isolates are a subset of Caribbean isolates, and French Guiana isolates are a subset of South American isolates. With the exception of *S. lycopersicum* (tomato) and *S. tuberosum* (potato), the host of isolation is shown collapsed to family level. Lower level taxonomic classifications are indicated with greater color saturation, and missing metadata is shown in white. b) Global distribution of the five major RSSC phylotypes across geographic regions, colored as in a). Pie charts are logarithmically scaled by the count of isolates per region, with shading to indicate phylotype proportion. c) UpSet plot displaying breadth of host of isolation for the four best sequenced lineages compared to the remainder of all other RSSC isolates. Host of isolation is colored as in a), and the sum of host species or families infected is shown on the right.

The global phylogeography of the RSSC reveals patterns of long-distance dissemination and epidemic expansion. Our analysis of 409 new whole-genome sequences from *Ralstonia* isolated from crops, weeds, soil, and freshwater environments during the outbreak paired with 407 genomes available on NCBI shows that diverse populations of both *R. solanacearum* (Rso) and *R. pseudosolanacearum* (Rps) are circulating in the Americas (Wicker et al. 2007, 2009; Deberdt et al. 2014). We inferred a maximum likelihood tree using a 137,423 bp SNP alignment obtained after core gene identification in the 816 RSSC genomes, followed by alignment generation and invariant site removal (Fig. 1; Fig. S1, Table S1). The outbreak documented in Martinique was caused by the expansion of two lineages from each species: Rps I-18 and Rso IIB-4NPB, consistent with MLSA showing the presence of both in Wicker et al. (2007). The center of origin of Rso and Rps is in the Americas and Asia, respectively (Wicker et al. 2012). Multiple intercontinental dissemination events of both Rso and Rps lineages are evident in the tree, including a global outbreak of Rso IIB-1 on potato (*S. tuberosum*) and the introduction of Rps lineages from Asia into the Americas (Fig. 1; Fig. S1), which likely have different geographic origins in Asia. These became well established in the Americas, where they have since diversified, primarily in solanaceous hosts.

Rps I-18 is widely distributed across Martinique, French Guiana, Brazil, and Peru (Fig. 1; Fig. S1). This lineage shares a recent common ancestor with another lineage referred to as Rps I-14, also present in French Guiana, Guatemala, Costa Rica, and Colombia. The Rps I-14 sequevar is polyphyletic: a distantly related group of strains referred to by the same name is also circulating in French Guiana, along with members of Rps I-17 and I-47/I-48 (Fig. 1). Although a single dissemination event could be responsible for the introduction of a diverse pathogen population, it is more likely that multiple introductions of Rps occurred from different regions in Asia. The presence of Rps I in the Americas was documented as early as 1967 (Prior and Steva 1990); this work shows that Rps I-14 and I-18 are now well established in the Americas, where they have since diversified in primarily solanaceous hosts.

**Fig. 2.**
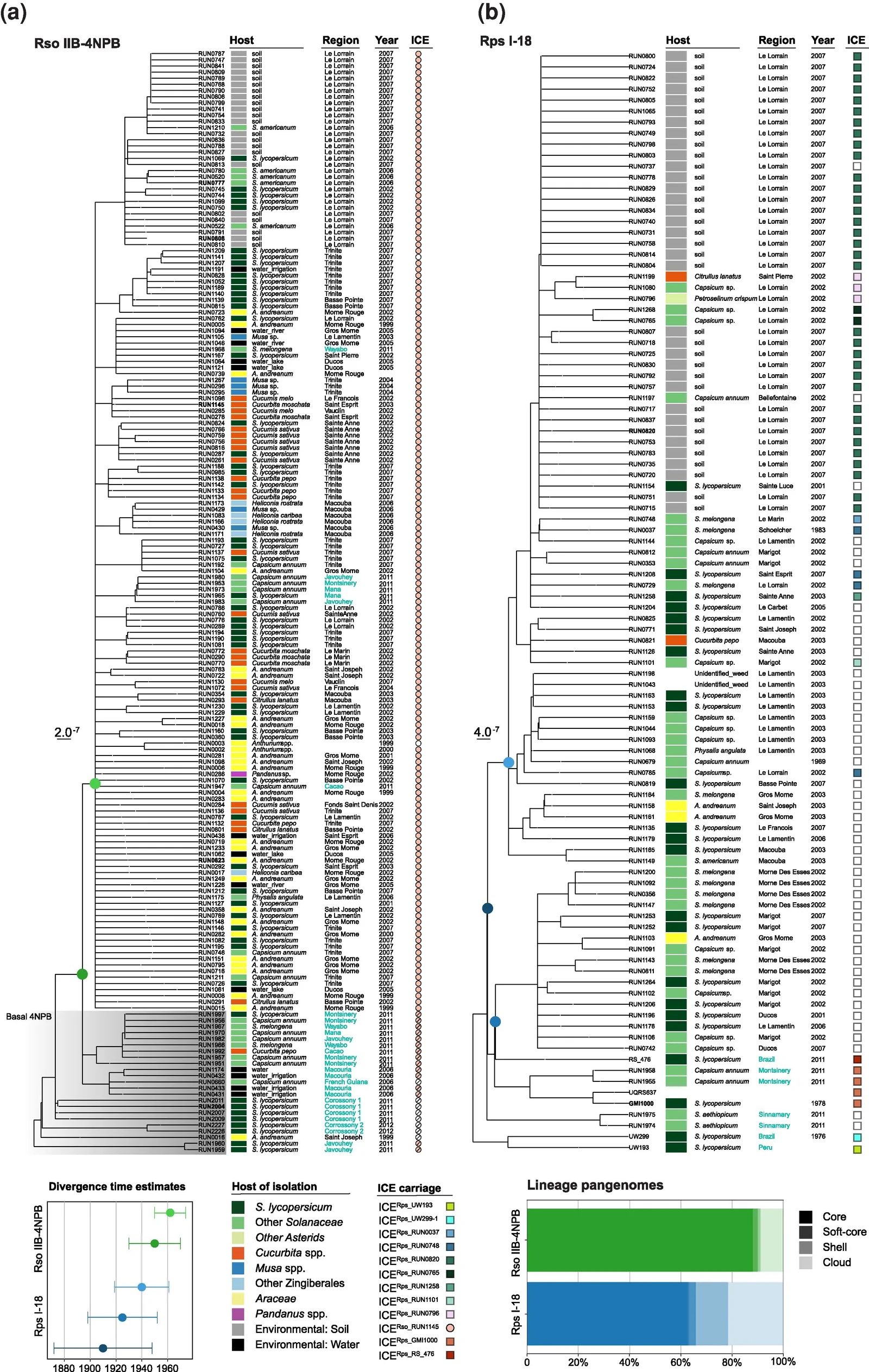
Outbreak lineage core genome trees. a) Rso IIB-4NPB maximum likelihood tree inferred from 5,206,677 bp nonrecombinant nonmobile core genome alignment (195 SNPs and 73 parsimony informative sites) using RUN1145 as reference. b) Rps I-18 maximum likelihood tree calculated from a 4,760,272 bp nonrecombinant core genome alignment using GMI1000 as reference with 311 SNPs and 116 parsimony informative sites. UQRS637 was suppressed on NCBI by the uploader so the metadata was removed from the tree. Internal nodes with bootstrap value above 80 are indicated with black circles in both a) and b). Strain labels shown in boldface in each tree indicate complete assemblies generated in this study and/or used as reference. ICEs are colored by type as in Fig. 4b (see below) with a diagonal line for basal Rso IIB-4NPB strains. Divergence time estimates of indicated nodes display estimated times and error bars derived from dated trees. The pangenome bar chart displays the proportion of core (present in 99% to 100% of genomes), soft-core (95% to 99% of genomes), shell (15% to 95% of genomes), and cloud genes (present in less than 15% of genomes) identified with Panaroo for each lineage pangenome.

In contrast to Rps, Rso is endemic and has been diversifying in the Americas for some time. Although the outbreak in Martinique was caused by the near-parallel expansion of Rso IIB-4NPB and Rps I-18, these are highly clonal (Fig. 2). The Rso IIA isolates encompass the greatest phylogenetic diversity in the Martinique and French Guiana collection. The Rso IIA are broadly distributed across the Americas and are primarily isolated from tomato (68%), but display little evidence of recent epidemic expansion (Fig. 1; Fig. S1). Rso IIB lineages are responsible for both regional and global outbreak events however, most notably on banana (Rso IIB-4) and potato (Rso IIB-1). The potato outbreak lineage Rso IIB-1 (formerly R3bv2) has been isolated from other *Solanum* spp., *Pelargonium* spp. (formerly *Geranium* spp.), surface water, and soil. Although the center of origin of Rso IIB-1 appears to be in South America, its rapid global expansion indicates that international trade in seed potatoes is the likeliest mechanism of long-distance dissemination, highlighting the risk of rapid transmission during the movement of infected plant material (Clarke et al. 2015; Abdurahman et al. 2017; Dewberry et al. 2024; Tessema et al. 2024).

Outbreaks of Rso on banana are well documented in the Americas, where it is referred to as Moko disease. Moko disease is clearly polyphyletic: regional outbreaks in Panama, Costa Rica, Colombia, Peru, Brazil, and French Guiana have been caused members of three Rso IIB and four Rso IIA lineages from at least the 1960s (Fig. 1; Fig. S2) (Albuquerque et al. 2014; Lowe-Power et al. 2022). Since banana was only introduced to the Americas as a crop in the 16th century (Price 1995), it is a matter of some interest that the most deeply branching basal members of Rso IIB are isolated from *Musa* spp. along with *Heliconia* spp. and *Anthurium* spp., both endemic to the Americas (Fig. 1; Fig. S2) (Kress 1984; Croat 1988). The expansion of high-density single-variety banana monocultures likely increased opportunities for the pathogen to emerge and spread in agricultural areas, in turn increasing likelihood of spread to other crop varieties, both endemic and introduced.

### Epidemic Expansion of Two Lineages After Their Dissemination From South America

The species complex tree confirms the surge in plant infections in Martinique is due to the recent clonal expansion of two lineages across the island: Rso IIB-4NPB and Rps I-18 (Fig. 1). Rso IIB-4NPB was isolated from the most diverse group of naturally infected hosts ever documented for a single lineage in *Ralstonia* to date, while Rps I-18 is primarily isolated from solanaceous hosts in the field (Wicker et al. 2007, 2009). We investigated the origins of each lineage by generating well-resolved, nonrecombinant core genome trees including closely related strains from mainland populations (Fig. 2). The Rso IIB-4NPB maximum likelihood tree was inferred from a 5,206,677 bp nonrecombinant nonmobile core genome alignment (195 SNPs and 73 parsimony informative sites) using Rso IIB-4NPB RUN1145 as the reference, while the Rps I-18 tree was inferred from 4,760,272 bp nonrecombinant core genome alignment (311 SNPs and 116 parsimony informative sites) using Rps I-18 GMI1000 as reference.

Basal members of Rso IIB-4NPB were primarily isolated from solanaceous hosts or freshwater sources on the South American mainland (Fig. 2; Figs. S2 and S3). The presence of basal Rso IIB-4NPB in Martinique (RUN0016) indicates that the basal French Guiana isolates are likely representative of the ancestral mainland population from which Rso IIB-4NPB emerged. The derived sublineage of Rso IIB-4NPB underwent limited spread on the mainland prior to its dissemination to Martinique. The derived sublineage exhibits limited genetic diversity, differing by only 166 nonrecombinant SNPs in the core genome. The presence of both basal and derived Rso IIB-4NPB on *Anthurium andraeanum* in Saint Joseph suggests this location and host are linked with the initial introduction of the novel lineage to Martinique (Fig. 2). Notably, Rso IIB-4NPB was also isolated from symptomatic banana in Trinité, indicating the appellation “nonpathogenic on banana” (NPB) for the lineage may not be wholly accurate.

The evolutionary history of Rps I-18 parallels that of Rso IIB-4NPB, as Martinique Rps I-18 was similarly derived from a broadly distributed mainland population (Fig. 2; Fig. S4). The Martinique lineage also displays limited diversity in the core genome and Type 3 secreted effector (T3E) repertoire, consistent with its recent epidemic expansion (Fig. 2; Fig. S1). Isolation from solanaceous hosts is highly lineage dependent and occurs with higher frequency among Martinique Rps I-18 than Rso IIB-4NPB (Fig. 2; Fig. S5). Spatial population structure is evident for Rps I-18 and Rso IIB-4NPB alike: the Rso IIB-4NPB and Rps I-18 isolates from Le Lorrain both form distinct and well supported clades in their respective trees, regardless of whether they were isolated from soil, cultivated, or wild *Solanum* spp. (Fig. 2).

We used these well-resolved nonrecombinant trees to separately infer divergence times for both Rps I-18 and Rso IIB-4NPB (Fig. 2). BactDating showed there is some correlation between the sampling date and root-to-tip distance for Rso IIB-4NPB (*R*^2^ = 0.16, *P* < 0.0001) and a permutation analysis showed the estimated divergence time outperformed random sampling in 77.6% of simulations (Fig. S6). Though weakly supported, the estimated divergence time for the split between the basal mainland and derived Martinique population of Rso IIB-4NPB is between 1930 and 1970 (Fig. 2; Fig. S6). Rps I-18 exhibits little temporal signal (*R*^2^ = 0.05, *P* = 0.04), yet date permutation analysis showed the estimated divergence time outperformed random sampling in 100% of simulations. The estimated divergence time for Rps I-18 is earlier, between 1870 and 1940 (Fig. 2; Fig. S7). During pangenome analyses, we found both Rps I-18 and Rso IIB-4NPB have similar numbers of core and soft-core genes (4,565 for Rps I-18 and 4,792 for Rso IIB-4NPB), but different accessory genome sizes: 487 accessory genes in Rso IIB-4NPB and 2,323 in Rps I-18 (Fig. 2, Tables S2–S4). While it is possible the difference in accessory genome sizes (amounting to 10% and 35% of the Rso IIB-4NPB and Rps I-18 pangenome, respectively) may be caused by differences in the rate or maintenance of horizontal acquisition events, this may also reflect different introduction times to Martinique. The data cumulatively indicates Rps I-18 was introduced to Martinique prior to Rso IIB-4NPB.

### Ancestral Changes in Virulence and Metabolic Gene Repertoires

**Fig. 3.**
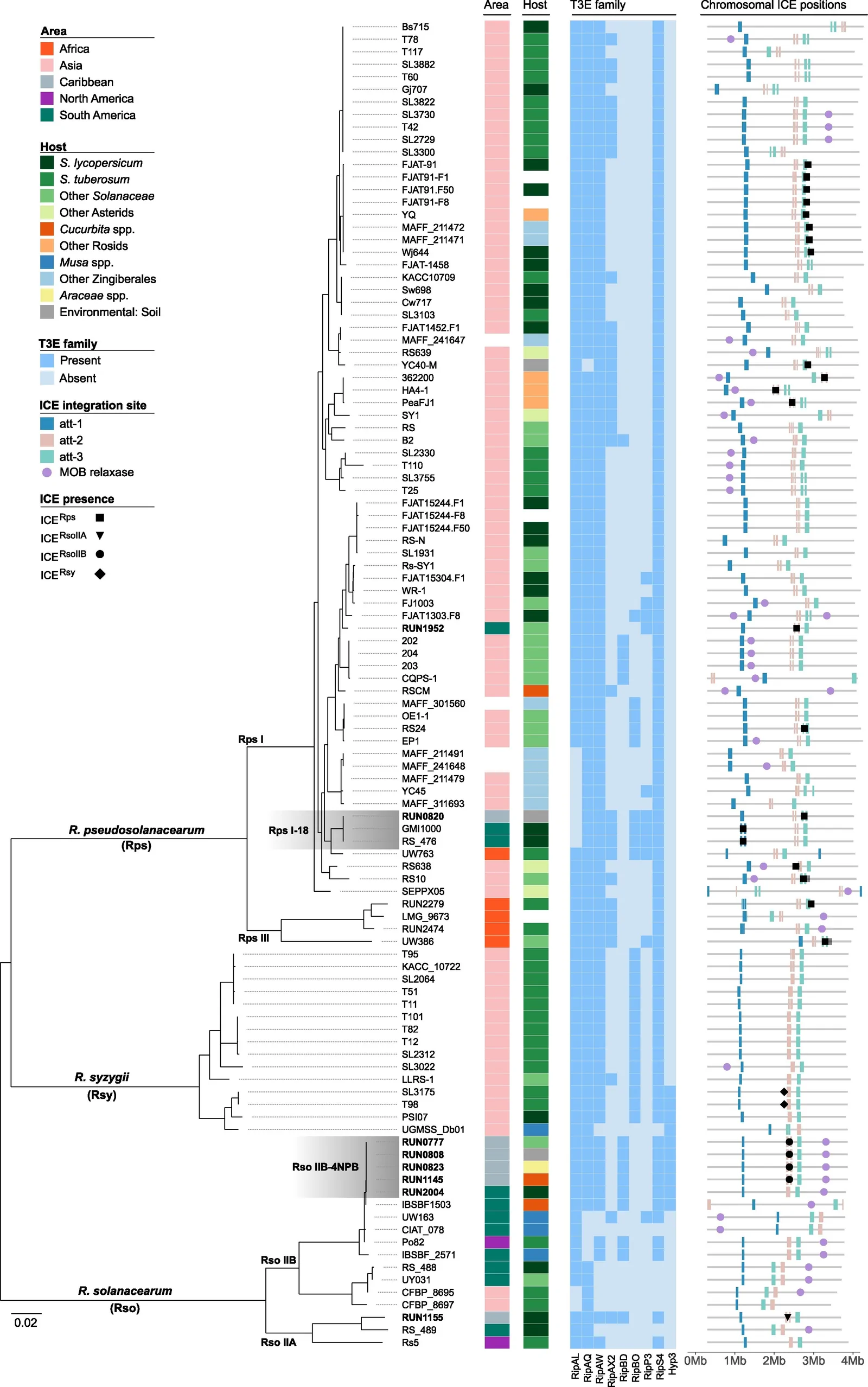
Virulence gene and ICE distribution across the *R. solanacearum* species complex. Maximum likelihood tree of all completely assembled RSSC genomes available in this study (*n* = 105). Strain labels shown in bold are complete or near complete assemblies generated in this study and/or used as mapping references. Geographic area and host of isolation are shown next to strain labels, followed by nine *Ralstonia* T3E families exhibiting presence/absence variation for Rso IIB-4 and Rps I-18 clades. Chromosomal ICE position displays the position of the ICE attachment sites on the main chromosome of each RSSC genome. The attachment sites are here defined as regions syntenic to the 50 kb up- and downstream flanking regions of the Rps I-18 GMI1000 (att-1, blue), Rso IIB-4NPB RUN1145 (att-2, pink), and Rso IIB-4NPB RUN1960 (att-3, green) attachment sites. Thin gray lines represent the length ranges of the chromosomes. MOB relaxases occurring in isolation (purple circles) are separately indicated from those adjacent to T4SS genes and forming putative ICEs (purple diamonds).

Despite the reduced genetic diversity and recent emergence of Rso IIB-4NPB, derived members of this lineage have been isolated from the widest variety of hosts during natural infections and display the broadest documented host range of any single lineage in the species complex (Fig. 2). Since the delivery of virulence proteins by the Type 3 secretion system is known to play an important role in successful host colonization and the repertoire of T3Es in turn is thought to play an important role in host specificity (Hueck 1998; Poueymiro et al. 2009; Landry et al. 2020), we identified the T3E repertoire across the entire dataset to determine whether the gain or loss of specific T3Es is linked with the emergence of Rso IIB-4NPB (Fig. 3; Fig. S1 and Table S5). Rso IIB-4NPB genomes display nearly uniform T3E repertoires, consistent with the recent expansion of this lineage, with no lineage-specific T3E changes compared to Rso IIB-4 (Fig. S2). Multiple T3E changes occurred in both the common ancestor of Rso IIB-4NPB and Rso IIB-4 and in Rso IIB-4 alone. For example, a single T3E is uniquely present in Rso IIB-4 and Rso IIB-4NPB, but absent in all other RSSC genomes: the hypothetical effector *T3E_Hyp13* (UW163_19700 in Rso IIB-4 UW163, OFBKGH_20220 in Rso IIB-4NPB RUN1145) (Fig. S1 and Table S5). Another T3E (*ripAX1*) is sufficiently divergent from other members of the effector family to be classified as a clade-specific gene by Panaroo pangenome analysis. Two effectors are present in Rso IIB-4 and absent in Rso IIB-4NPB: *ripAX2* and *ripP3* (Figs. S1 and S2 and Table S5). The effector *ripAX2* has a broad distribution across Rso IIA and IIB genomes and was therefore likely lost in Rso IIB-4NPB, while *ripP3* has a patchy distribution and was likely acquired by Rso IIB-4. Four effectors appear to have been lost in basal Rso IIB-4: *ripAQ*, *ripAW*, *ripBD*, and the predicted *T3E_Hyp3* effector. The loss of *ripAQ* is most striking as the family is otherwise present in all other RSSC strains. Since these effectors are present among related clades and/or across the species complex, the most parsimonious explanation for the pattern observed is they were lost in the ancestor of banana-infecting Rso IIB-4 strains rather than being acquired by ancestral Rso IIB-4NPB. The Rso IIB-4 isolated from monocot hosts (*Heliconia* spp., banana, and *Epipremnum* spp.) have more unique variants statistically associated with the host of isolation and exhibit more variation in effector repertoire than Rso IIB-4NPB (Fig. 3; Fig. S2 and Supplementary Results).

Our investigation of non-T3E acquisitions in the ancestor of Rso IIB-4NPB showed that 84 genes were acquired prior to the split between Rso IIB-4 and Rso IIB-4NPB (Table S6). These genes encode enzymes such as a TauD/TfdA family dioxygenase, shown to degrade phenoxy herbicides in soil-borne bacteria like *Burkholderia* spp. and *Cupriavidus necator* (formerly *Ralstonia eutropha*) (Suwa et al. 1996; Zaprasis et al. 2010; Newby et al. 2000; Füchslin et al. 2003; Bælum et al. 2006; Han et al. 2015); a CxxC motif protein (UW163_01710, OFBKGH_14170) associated with thiol-disulfide oxidoreductase activity and oxidative damage responses (Chivers et al. 1997; Quan et al. 2007); an electron acceptor flavodoxin (UW163_18870, OFBKGH_23185) involved in oxidative damage response (Sancho 2006); and a carboxymuconolactone decarboxylase in the protocatechuate catabolism pathway (UW163_18885, OFBKGH_23170), linked with lignin degradation and conversion of lignin-derived aromatic compounds to tricarboxylic acid intermediates like succinate and malate (Eulberg et al. 1998; Martim et al. 2024). In sum, although the function of many horizontally acquired genes remains unclear, ancestral acquisitions are often linked with the catabolism of aromatic compounds in soil or plant tissues.

### ICEs Are Circulating Between Co-occurring Pathogen Lineages

We explored whether Rps I-18 and Rso IIB-4NPB exchanged genetic material during their expansion across Martinique, since their co-occurrence in the same host or niche increases the likelihood of interspecies recombination. RECOPHY identified some regions with reduced (<10/kb) and elevated (120/kb) SNP density compared to the alignment-wide average of 53 SNPs/kb, yet distance trees for those regions display the same topology as the core genome tree, indicating homologous recombination between these lineages is unlikely to have occurred (Fig. S8) (Sakoparnig et al. 2021). ClonalFrameML analysis indicated that although the ratio of recombination to mutation in both lineages is low (R/theta = 0.159 for Rps I-18 and 0.152 for Rso IIB-4NPB), homologous recombination is occurring with unknown donors. Mobile elements constitute another means by which genetic material can be exchanged between bacterial pathogens. Pangenome exploration and association testing alike revealed the presence of ICEs present in co-occurring Rps I-18 and Rso IIB-4NPB, but absent in Rso IIB-4 (Fig. 3). The presence of related ICEs was also clearly supported by the gene and kmer-based association testing, as 26/27 genes associated with isolation in Martinique are ICE genes and all 83 soil-associated and 625/2,367 Martinique-associated kmers map to ICE genes (Figs. S9 to S11 and Tables S9 to S12).

**Fig. 4.**
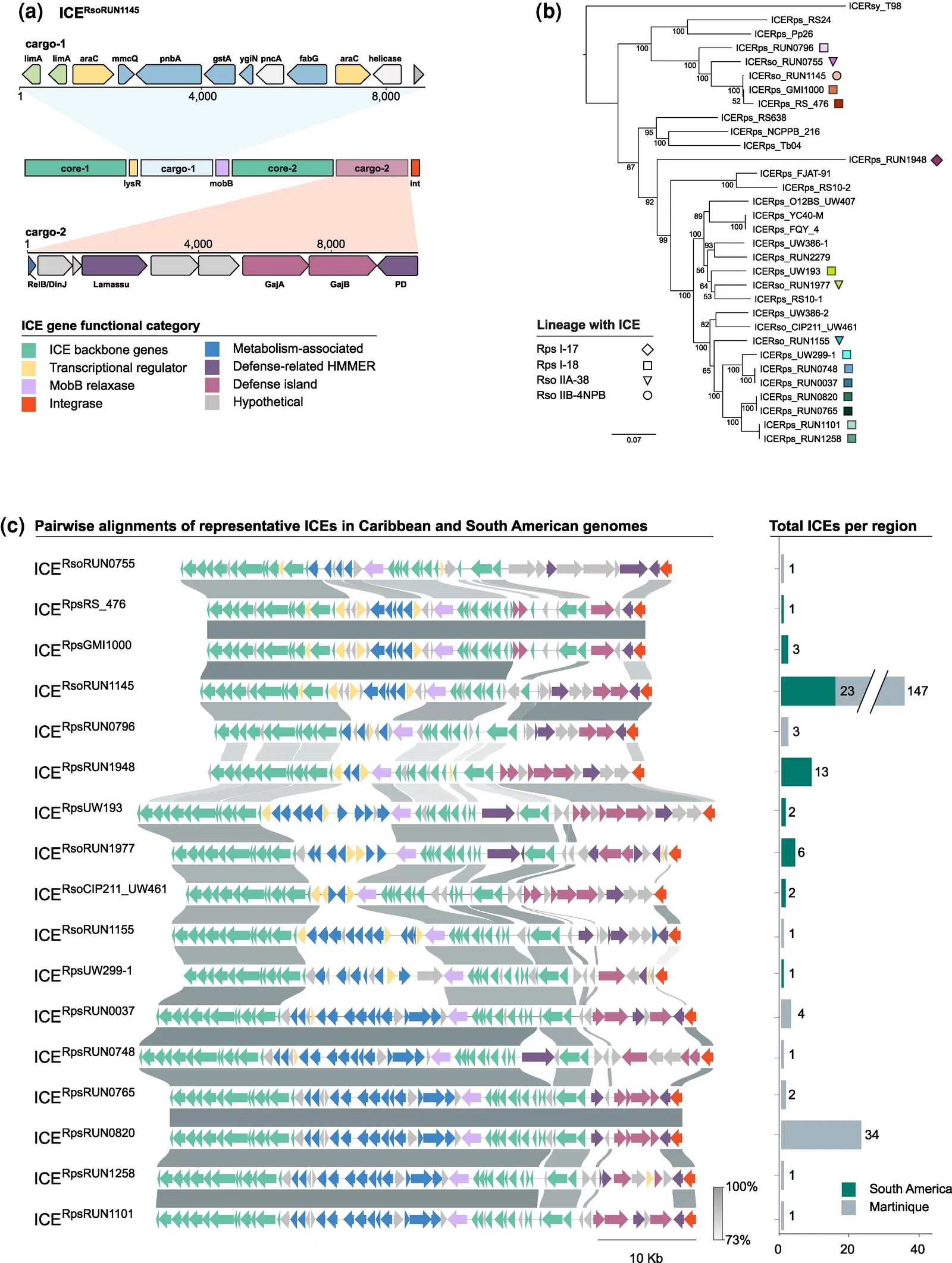
Genetic organization and diversity of *Ralstonia* spp. ICEs. a) Core and cargo gene arrangement in the Rso IIB-4NPB ICE^RsoRUN1145^. Genes are colored according to their functional category. b) RAxML likelihood tree inferred from core-1 genes in 34 nonredundant RSSC ICEs. Icons are shown next to ICEs circulating in Martinique and French Guiana. c) Pairwise MUMmer alignments of nonredundant Caribbean and South American ICEs. Genes are colored according to functional category. The total number of genomes carrying each ICE and their geographical origin are shown in the stacked bar chart to the right of each ICE.

Since the emergent Rso IIB-4NPB lineage acquired an ICE absent in Rso IIB-4 and related ICE genes were shared between both Rps I-18 and Rso IIB-4NPB in Martinique, we first used all available complete genomes (n = 133) to predict ICEs in the RSSC and define their integration sites in the genome. We found a total of 30 ICEs in the complete genomes, of which 17 lack direct repeats but satisfy other criteria for ICE identification (presence of T4SS gene clusters, a *mob* relaxase and integrase sequence) (Fig. 3). Some genomes have *mob* relaxase genes flanked by direct repeats but lack a proximal T4SS: these may be degenerate ICEs or integrative mobilizable elements (IMEs) (Table S13). Each genome typically carries a single ICE integrated into one of three preferred chromosomal integration sites, though UW386 and RS10 carry two ICEs. All ICEs identified in complete RSSC genomes are located on the main chromosome. We then expanded our search across all genomes in the dataset and used alignments and tree building to identify a set of 34 nonredundant ICERs representing a total of 280 complete ICEs in 97 *R. pseudosolanacearum*, 181 *R. solanacearum*, and 2 *R. syzygii* genomes. Three isolates carry two distinct ICEs each (Rps I UW299, Rps RS10, and Rps III UW386), while the remainder carry single ICEs. At the global scale, most ICE diversity is harbored in Rps I (23/34 nonredundant ICEs), followed by Rso (5/34), Rps III (4/34), and Rsy (1/34) (Fig. 3; Table S14). ICE^RsoRUN1145^ carriage is nearly ubiquitous among Rso IIB4-NPB isolates: identical copies of ICE^RsoRUN1145^ are found in 170 Rso IIB-4NPB isolates from both Martinique and French Guiana, though basal Rso IIB-4NPB isolates typically lack the ICE (Fig. 2). Although fewer Rps I-18 were isolated in Martinique and 43% of these are ICEless, Rps I-18 carries 11 unique ICEs compared to only in Rso IIB-4NPB (Figs. 2 and 4). Some Rps ICEs of the same type vary in the presence of insertion sequences (ICE^RpsRS10–1^) or SNPs and indels (ICE^RpsRUN0037^, ICE^RpsRUN0820^) (Table S14).

*Ralstonia* ICEs have a bipartite structure, with two core gene clusters separated by two distinct hotpots of cargo gene integration (Fig. 4). The first core gene cluster (core-1) includes the conjugal transfer genes and exhibits less variation in gene content compared to the second core gene cluster (core-2). The average pairwise nucleotide identity of core-1 sequences from all 27 nonredundant ICEs is nevertheless very low (83.4%), indicating *Ralstonia* spp. ICEs carried by closely related strains can have very distant evolutionary histories. Strikingly, all *Ralstonia* ICEs display consistent functional differentiation in their hotspots of cargo gene integration: the first hotspot (cargo-1) always carries genes whose functions are related to metabolism, while the second hotspot (cargo-2) encodes bacterial defense-related genes (Fig. 4; Fig. S12).

**Fig. 5.**
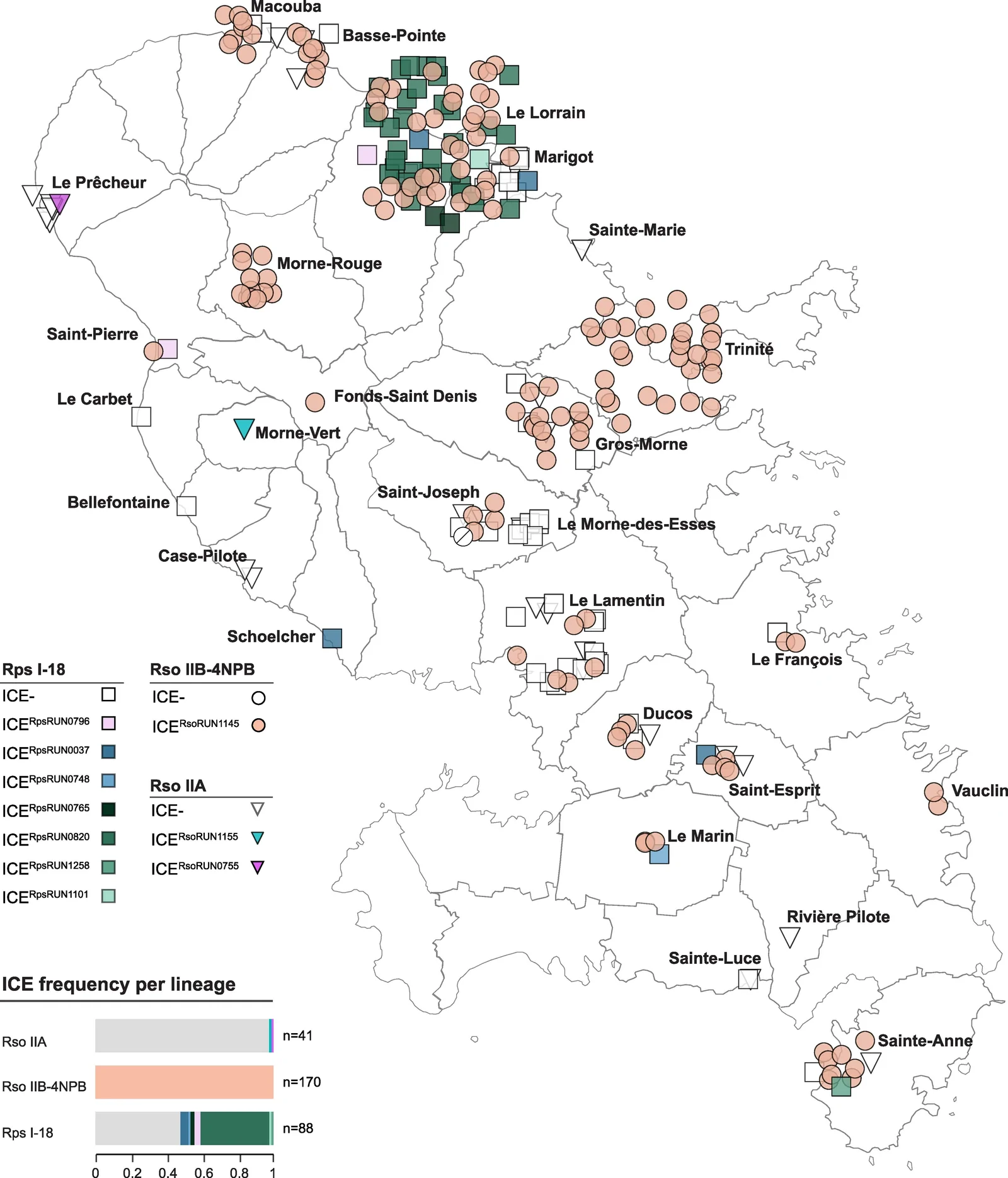
ICE diversity in outbreak lineages. Sampling location of all Martinique isolates sequenced in this study, with longitude and latitude jittered for visualization purposes. Icon shape and fill indicate strain lineage and ICE type as in Fig. 4b with a diagonal line for basal Rso IIB-4NPB strains. The frequency of ICE type carriage by lineage is shown inset.

A likelihood tree inferred from the first cluster of core genes (core-1) shows that the Rso IIB-4NPB ICE^RsoRUN1145^ shares a common ancestor with two Rps I-18 ICEs circulating on the mainland: ICE^RpsGMI1000^ and ICE^RpsRS_476^ (Fig. 4). The Rps I-18 ICEs ICE^RpsGMI1000^ and ICE^RpsRS_476^ have identical cargo but share only 98.7% pairwise identity in core-1, whereas Rso IIB-4NPB ICE^RsoRUN1145^ and Rps I-18 ICE^RpsGMI1000^ vary in defense cargo-2 but share 99.8% identity in both core and metabolic cargo-1 gene clusters. In contrast, the core gene pairwise identity of Rso IIB-4NPB RUN1145 and Rps I-18 GMI1000 is 90.2%, indicating the ICEs share far more recent common ancestry than the bacterial strains in which they are found. It is clear ICE^RsoRUN1145^ was acquired by basal members of the emergent Rso IIB-4NPB lineage (Fig. 3a), whereas Rps I-18 carries 13 different ICEs including ICE^RpsGMI1000^ (Fig. 3b). Visualization of the spatial structure of ICE diversity among the densely sampled Martinique populations shows that there are local hotspots of ICE carriage and exchange in Martinique (Fig. 5). Most of this variation is present in a single site in Martinique (Le Lorrain), where 40 Rps I-18 isolates carry one of four different ICEs (types). Only one Rps I-18 in Le Lorrain is ICEless, while the majority (43/49) of Rps I-18 sampled elsewhere in Martinique are ICEless. Although every one of the 35 Rps I-18 isolated from soil were sampled in Le Lorrain, isolation from soil alone does not explain this pattern of ICE carriage since all 22 Rso IIB-4NPB isolated from soil in Le Lorrain carry ICE^RsoRUN1145^. ICE carriage similarly varies by site and host strain lineage on the mainland (Fig. S13). There is a single site in French Guiana where both ICE^RpsGMI1000^ and ICE^RsoRUN1145^ are circulating in Rps I-18 and Rso IIB-4NPB (Montsinery).

The metabolic cargo carried by ICE^RsoRUN1145^, ICE^RpsGMI1000^, and ICE^RpsRS_476^ includes genes linked with detoxification, such as limonene 1,2-epoxide hydrolases *limA* (Barbirato et al. 1998) (Rso RUN1145 OFBKGH_09550, OFBKGH_09555); a phthalate ester hydrolase *pnbA*; an antibiotic biosynthesis monooxygenase *ygiN* (also known as ABM, OFBKGH_09580); glutathione S-transferase *gstA* linked with the detoxification of organochlorine compounds (1-chloro-2,4-dinitrobenzene) used as an herbicide and pesticides; and a pyrazinamidase/nicotinamidase *pncA*, which converts nicotinamide to nicotinic acid, and *fabG*, a reducing agent of ethyl 4-chloroacetoacetate, an intermediate used in herbicide, and pesticide production. ICE^RsoRUN1145^ differs in the carriage of antiviral Gabija and Lamassu defense systems; however, ICE^RpsGMI1000^ and ICE^RpsRS_476^ carry identical RloC and Thoeris systems (Doron et al. 2018; Cheng et al. 2021) (Fig. 4). The ICE^RsoRUN1145^ Gabija and Lamassu defense systems share 90.3% identity with a defense cluster in a different Rps I-18 ICE^RpsRUN0796^ sampled in Martinique.

### A History of Viral Encounter in the Emergent Lineage

The carriage of antiviral defense gene clusters on the ICE and presence of type I-E CRISPR-Cas systems among RSSC genomes indicates the plant pathogen is frequently challenged by viral attack. CRISPR-Cas systems confer immunity to phage via the acquisition of spacer sequences from prior infections. We found that both Rso IIB-4 and Rso IIB-4NPB share conserved CRISPR-Cas operons (*cas3*, *casA/cas8e*, *casB/cas11/cse2*, *cas7e*, *cas5e*, *cas6e*, *cas1e*, *cas2*) but vary in their CRISPR arrays. These genomes share 15 leader-distal spacers acquired prior to the split between IIB-4 and IIB-4NPB but differ in more recently acquired leader-proximal spacers (Fig. S14) (He and Deem 2010; Da Silva Xavier et al. 2019). Far more spacers were recently acquired by Rso IIB-4NPB after its dissemination to Martinique: Rso IIB-4NPB CRISPR arrays are nearly ten times longer (171 spacers) than the Rso IIB-4 arrays (20 spacers). We used spacer sequences as queries for INPHARED phage database searches and identified six phages targeted by Rso IIB-4NPB alone (Table S15). Interestingly, five of these are phages known to infect Rps I (RPZH3, RS603, RSMSuper, Eline, and Raharianne), indicating the co-occurring lineages encounter the same viral threats, though potentially with different CRISPR-mediated outcomes (Gonçalves et al. 2021; Trotereau et al. 2021; Lin et al. 2023). The new IIB-4NPB lineage also acquired three *Caudoviricetes* prophages compared absent in Rso IIB-4 (Fig. S15, Table S16). Two of these are present in other Central and South American Rso isolates, while the third had not previously been identified in *R. solanacearum*, but has BLAST hits to *Ralstonia* metagenome-assembled genomes from estuary sediment in China and a Brazilian sugarcane pathogen *Xanthomonas albilineans* (Fig. S16). The presence of multiple defense systems, amplified CRISPR arrays, and prophage acquisition events all attest to the importance of antiviral defenses for the successful expansion of Rso IIB-4NPB across soil, water, and plant microbiomes in Martinique.

## Discussion

Our knowledge of the evolutionary and ecological processes acting on pathogen populations during outbreak events is typically limited by inadequate sampling within and beyond the focal host. This constrains our efforts to infer outbreak origins and identify the mechanisms remodeling pathogen genomes prior to outbreak events. Dense sequencing of a collection of isolates sampled across a wide range of agricultural hosts and environmental reservoirs provides us with unique insight into the processes and mechanisms acting on plant pathogenic *Ralstonia* spp. The collection captured the parallel expansion of both *R. solanacearum* IIB-4NPB and *R. pseudosolanacearum* I-18 during the first documented multilineage outbreak of a bacterial plant pathogen. Though at present rare (Abou Fayad et al. 2024), the detection of multilineage outbreaks will likely increase with greater sampling and sequencing efforts.

Our work shows the island population of Rps I-18 diverged from the mainland population 115 (±40) years ago and preceded the arrival of Rso IIB-4NPB in Martinique. Rso IIB-4NPB emerged more recently, diverging from a common ancestor on the mainland approximately 75 (±20) years ago. Basal members of the Rso IIB-4NPB lineage spread to multiple sites in French Guiana both prior and subsequent to the emergence of the sublineage responsible for the outbreak in Martinique. Although divergence dates are not equivalent to dissemination dates, Rso IIB-4NPB is likely to have arrived in Martinique after 1960 (±10), where it was first detected after undergoing epidemic expansion across a wide range of plant hosts.

The Rps I-18 lineage retains a clear signature of specialization on the Solanaceae family, while the Martinique Rso IIB-4NPB lineage, which has an average pairwise identity of 99.998%, is unique in being isolated from most diverse group of hosts ever documented for a single lineage in the field. Since basal Rso IIB-4NPB are primarily associated with *Solanaceae* infections, we explored whether the broad host-range phenotype was associated with the acquisition of T3Es, known to promote virulence by manipulating host defenses. We found little genomic evidence of T3E innovation in Rso IIB-4NPB, which differs from Rso IIB-4 by a single hypothetical effector gain (*T3E_hyp3*) and two losses (*ripAX2* and *ripP3*). It is possible that regulatory changes or allelic variation alter Rso IIB-4NPB host range (Ailloud et al. 2016), but the observed differences appear unlikely to account for the apparent expansion in host-range compared to Rso IIB-4, particularly since single effector deletions typically have limited consequences for pathogenicity. The genomic evidence shows instead the acquisition of metabolic and antiviral resistance genes repeatedly occurred in the ancestor of Rso IIB-4NPB, highlighting their significance for the successful establishment of the sublineage in a wide range of hosts and environments.

Bacterial metabolism is increasingly recognized as an important component of pathogenicity in mammalian and plant pathogens alike (Eom et al. 2015; Freyberg and Harvill 2017; Rowley and Kendall 2019; Cai et al. 2023). *Ralstonia* spp. can assimilate multiple carbon and nitrogen sources commonly available in xylem sap and degrade phenolic compounds produced during host defense responses (Lowe-Power et al. 2016; Baroukh et al. 2022, 2023 June 21; Hamilton et al. 2024). Metabolism is regulated according to infection stage (De Pedro-Jové et al. 2021) and serves as an important target of selection during experimental evolution on a novel host (Gopalan-Nair et al. 2021). Rps I-18 GMI1000 also exhibits growth on and chemotaxis toward malate, an organic acid available in the xylem and soil alike (Baroukh et al. 2022). The emergent Rso IIB-4NPB sublineage also acquired multiple genes whose products are linked with the degradation of environmental pollutants, reactive xenobiotics, and plant-derived metabolites. Homologs of genes carried by the ICE^RsoRUN1145^ are variously linked with the detoxification of a plant secondary metabolites (limonene 1,2-epoxide hydrolase *limA*) (Rinaldi et al. 2018), catalysis of nicotinamide (pyrazinamidase/nicotinamidase *pncA*), and degradation of environmental pollutants like phthalate esters (phthalate ester hydrolase *pnbA*) (Ding et al. 2015). A *tauD*/*tfdA* family dioxygenase acquired by homologous recombination in the ancestor of Rso IIB-4NPB may also confer a growth advantage in environments exposed to phenoxy herbicides. The ability to metabolize soil pollutants is documented among relatives of plant pathogenic *Ralstonia* spp. like *Cupriavidus necator* (formerly *R. eutropha*), which is known to degrade the herbicide 2,4-dichlorophenoxyacetic acid (2,4-D) (Nicolaisen et al. 2008; Ledger et al. 2009; Papade et al. 2025). The acquisition of novel or more efficient metabolic enzymes may allow *Ralstonia* spp. to resist oxidative stress and use more carbon and energy sources in plant tissues and soil environments, fueling faster growth and enhancing plant colonization.

Emerging pathogens also serve as hosts for potential parasites. ICE carriage of multiple defense systems, Rso IIB-4NPB acquisition of multiple prophages, and the considerable expansion in CRISPR repeats attest to the importance of bacterial immunity for pathogen success. Interestingly, phage resistance has been similarly implicated in the success of the seventh pandemic lineage of *Vibrio cholerae* (LeGault et al. 2021; Adams et al. 2025). Remarkably, we found that Rso IIB-4NPB CRISPR spacers with identifiable origins correspond to phage known to successfully infect *R. pseudosolanacearum* strains like Rps I-18, indicating the parallel expansion of both Rps I-18 and Rso IIB-4NPB resulting in shared exposure to lytic phage. A multilineage outbreak also provides opportunities for the exchange of bacterial immunity genes via the movement of ICEs however, as we found here.

ICEs are highly effective agents of horizontal exchange between even distant relatives (Colombi et al. 2024). Their ability to transfer entire functional genetic modules in a single step allows for rapid adaptation to novel hosts and environmental niches (Colombi et al. 2017). The presence of ICEs among all *Ralstonia* spp. has clear implications for pathogen evolution in the field and may have been pivotal for the success of Rso IIB-4NPB in Martinique. We found that *Ralstonia* ICEs display a bipartite structure, with alternating core and cargo gene integration hotspots that clearly exhibit specialization in accessory gene function. Despite having variable gene content, *Ralstonia* spp. ICE cargo-1 always carries metabolism-associated genes and cargo-2 always carries bacterial immunity genes. Although site-specific integrases targeting each hotspot must be implicated, it is not clear how specialization in gene function rather than gene content can be maintained over long evolutionary distances and in different bacterial host genotypes.

We found species-specific variation in ICE diversity and spatial hotspots of ICE carriage across Rps I-18 and Rso IIB-4NPB isolated in Martinique. At the global scale, ICE carriage is more common among *R. pseudosolanacearum*, where 23/34 nonredundant ICEs are found. This is similarly the case in Martinique, where Rps I-18 harbors considerably more ICE diversity despite having a lower frequency of ICE carriage. It is necessary to be cautious when drawing conclusions regarding ICE prevalence and diversity based on limited sequencing, since ICE carriage evidently varies from one sampling site to another within one lineage but not the other. The rapid movement of ICEs prevents reliable inference of the source and directionality of transfer events, but their absence from Rso IIB-4, near ubiquity of a single ICE in Rso IIB-4NPB and its shared ancestry with Rps ICEs cumulatively suggest ICE^RsoRUN1145^ was acquired by Rso IIB-4NPB from a larger pool of mobile elements circulating among Rps on the mainland. It is not possible to determine the precise time, location, or identity of the donor strain from which ICE acquisition occurred in emergent Rso IIB-4NPB. Whether directly or via some intermediary, ICE transfer can evidently occur both within and between *Ralstonia* species however. In this case, ICEs may have moved from introduced lineages of *R. pseudosolanacearum* into endemic *R. solanacearum*. Genetic exchange between endemic and introduced *V. cholerae* has been documented (Dorman et al. 2020; Lassalle et al. 2023), but has not been previously demonstrated for a bacterial plant pathogen. Since multiple accessory genes are introduced in a single step, ICE transfer can have dramatic and immediate effects on recipient strain fitness and pathogenicity. This underscores the need for field surveillance strategies that extend beyond focal pathogen populations to the broader community and reservoir of mobile elements that fuel their evolution. As agricultural intensification continues to reshape plant–microbe interactions, pathogen lineages that can rapidly acquire and integrate novel genetic material are more likely to exploit novel niches and hosts.

## Materials and Methods

### Genome Sequencing

*Ralstonia* spp. were collected in Martinique and French Guiana as described in Wicker et al. (2009) and Deberdt et al. (2014). Isolates collected after the initial sampling were assigned to phylotype and sequevar by sequencing endoglucanase (*egl*) and mutation mismatch repair (*mutS*) genes. For this study, we extracted genomic DNA from 487 strains using Promega Wizard, then used a modified Nextera protocol for library preparation (Meier et al. 2021). Paired-end sequencing with Illumina NextSeq 2000 was then performed. Since the first round of sequencing produced under 25-fold average coverage and under 40-fold maximum coverage, a second round of library preparation and sequencing was performed. Eight isolates (RUN0777, RUN0808, RUN0820, RUN0823, RUN1145, RUN1155, RUN1952, and RUN2004) were additionally sequenced using Oxford Nanopore Technology with a FLO-MIN106 flow cell and SQK-RBK004 barcoding kit. Basecalling and demultiplexing was performed with guppy_basecaller (v. 5.0.7) and guppy_barcoder v. 4.5.4 (Oxford Nanopore Technologies).

### Genome Assembly and Annotation

Paired-end 150 bp reads of both Illumina sequencing runs were filtered and trimmed with fastq_screen (Wingett and Andrews 2018), bbduk (Bushnell 2024), and fastp (Chen et al. 2018, 2023) (–dedup –detect_adapter_for_pe –cut_front 15 –length_required 50). Assembly was performed with SPAdes (Bankevich et al. 2012; Prjibelski et al. 2020) using both separate and pooled (ie both sequencing runs) read sets. QUAST was used for assembly quality assessment (Gurevich et al. 2013). Pooling both sequencing runs improved average assembly N50 values from 73 to 195 kb and decreased average contigs per assembly from 370 to 217, so assemblies generated from pooled sequencing runs were used. After filtering out assemblies with under 50-fold coverage, over 250 contigs, total assembly length exceeding 6 Mb and N50 exceeding 75 kb, a total of 409 assemblies were retained for downstream analyses.

Oxford Nanopore long-read data was filtered with Filtlong (v. 0.2.1) (Wick) to remove reads under 1Kb and the 5% of reads with the lowest quality values, then assembled with Trycycler (v. 0.5.4) (Wick et al. 2021), using Flye (Kolmogorov et al. 2019), Miniasm (Li 2016) with Minipolish (Wick and Holt 2022), and Raven (Vaser and Šikić 2021) as underlying assemblers. Contigs that did not pass the reconciling step of Trycycler pipeline were manually removed. Final Trycycler consensus sequences were polished three times using long reads with Medaka (Nanoporetech), followed by two rounds of short read polishing using BWA (Li 2013; Vasimuddin et al. 2019) and Polypolish (Wick and Holt 2022), using separate batches of the Illumina reads produced by each sequencing run.

All assemblies were annotated by Bakta (Schwengers et al. 2021) using Prodigal (Hyatt et al. 2010) gene models and a reference protein database built using all complete *Ralstonia* spp. genome assemblies available from the RefSeq database in October 2022.

### NCBI Datasets and Reference Genomes

All available *R. pseudosolanacearum, R. syzygii*, and *R. solanacearum* genome assemblies were downloaded from NCBI in February 2023 to provide additional phylogenetic context for analyses. Assemblies with fewer than 200 contigs, total assembly length between 5.0 and 6.2 Mb, N50 over 75 Kb, and unflagged by the NCBI Genome (Sayers et al. 2022) for evidence of potential contamination were retained. A total of 410 *Ralstonia* spp. genomes passed filter (Table S1). Three isolates were present in duplicate in the NCBI filtered dataset (MAFF 211479, UW163, and UW551), and the lowest quality duplicate assembly was removed, producing a final NCBI dataset of 407 assemblies. These were then reannotated with Bakta and merged with the 409 assemblies produced in this work for downstream pangenome analyses. Metadata (host, geographical region and year of isolation) for genomes in the NCBI dataset was obtained by inspecting Biosample entries. If the information was missing in Biosample entries, we used RSSCdb records (Lowe-Power et al. 2022) or publications describing the initial isolation of the strain of interest. Metadata for the strains CIP128_UW455, UW258 and NCPPB282_UW491 was not used for the visualization due to discrepancies. Presence of RSSC phylotypes in different geographic regions was visualized using matplotlib.

### Lineage Effects on the Frequency of Isolation From *Solanaceae*

We tested whether bacterial lineage (Rso IIA, Rso IIB-4NPB, or Rps I-18) determines the likelihood of isolation from a *Solanaceae* host on Martinique using χ² tests of independence with the chi2_contingency function from the scipy package. Contingency tables of isolation frequencies were derived using data for Rso IIA, Rso IIB-4NPB, and Rps I-18 isolation from *Solanaceae* and non-*Solanaceae* plants sampled on Martinique, excluding isolates from environmental sources or missing metadata. Tests were performed for two pairs (Rso IIB-4NPB vs Rps I-18 and Rso IIB-4NPB vs Rso IIA) to determine whether the probability of isolation from *Solanaceae* is independent of *Ralstonia* lineage. Observed and expected distribution was visualized using matplotlib. Python UpSet plots were created by applying the generate_counts function to the host of isolation categories (Table S1).

### T3E Identification

Amino acid sequences of 118 *R. solanacearum* T3Es from the manually curated RalstoT3E database (Peeters et al. 2013) were downloaded and used as query sequences for DIAMOND BLASTp (Buchfink et al. 2015) searches against proteomes of the Bakta-annotated assemblies, using the –ultrasensitive mode. Hits were filtered using a S30L25 strategy: retaining sequences with alignment identity of at least 30% and query sequence coverage of 25% or greater. Since the T3E query set includes multiple alleles of the same effector families, hits were then grouped into T3E families by sorting first by identity and query length coverage. T3E family assignment for each protein sequence was performed using the most significant T3E hit. Effector presence and absence was visualized by a heatmap in R, omitting duplication events. This approach was tested by comparing the predicted T3E repertoires in Rps I-18 GMI1000 and Rsy RPSI07 with the ones provided in the RalstoT3E database. Average pairwise similarity of T3E repertoires within RSSC clades was estimated by Scikit-learn function cosine_similarity (Pedregosa et al. 2011) using the information about presence–absence of effector families as an input binary matrix.

### Pangenome Inference and Core Gene Tree Reconstruction

Panaroo (Tonkin-Hill et al. 2020) was used to identify core and flexible genomes for the RSSC dataset, ie the merged dataset of 816 *Ralstonia* genomes sequenced in this study (409 strains) and downloaded from NCBI (407 strains). A concatenation of core genes was used as an input alignment for phylogenetic analysis of the species complex. Alignment columns with gaps or ambiguities and invariant sites were removed with SNPsites (Page et al. 2016), producing a final alignment of 137,423 bp. Tree inference was then performed using IQtree (Nguyen et al. 2015) with a GTR + ASC model of sequence evolution, model for alignments that do not contain constant sites, and 1,000 bootstrap replicates. Panaroo gene presence and absence tables for the RSSC dataset were queried using a custom Python script to identify genes uniquely present in either Rps I-18, Rso IIB-4, or Rso IIB-4NPB. Genes are referred to as clade-specific when they are present in at least 95% of all genomes in a given focal clade, and present in no more than ten nonfocal clade assemblies. The ggtree R package (Yu et al. 2017), matplotlib, and Python UpSetPlot were used for all tree and metadata visualization.

The species complex tree from the set of core genes identified by Panaroo was used for clade delineation and lineages assignment. Most genomes clustered according to the phylotypes initially assigned by *egl* and *mutS* sequencing in Wicker et al. (2007) and Deberdt et al. (2014), although some discrepancies are present. We found that previous sequevar designations can provide an inaccurate view of strain relatedness, eg RUN2182 is classified as a Rps I-48, but groups with Rps I-47 in the core gene tree. Some strains were also reclassified into different phylotypes based on the core gene tree and RUN1259 was initially classified as Rso IIB but is in fact Rso IIA (Fig. S1 and Table S1). The clade designation in this work may therefore differ from historically assigned sequevar or even phylotype labels, since these were first assigned using *egl* and *mutS* sequences rather than whole-genome sequencing and core gene tree reconstruction. The criteria for membership in a named clade are exceeding a minimum shared identity of 99% in the RSSC core gene alignment.

Separate pangenome analyses and core gene tree reconstructions were performed on three specific clades: *R. pseudosolanacearum* phylotype 1 sequevar 18 (Rps I-18, 102 strains), *R. solanacearum* phylotype 2 sequevar 4 (Rso IIB-4, 226 strains), and the emergent lineage *R. solanacearum* phylotype 2 sequevar 4 NPB (Rso IIB-4NPB, 180 strains). The strains forming each clade were identified with reference to the RSSC core gene tree (Fig. 1). Assemblies for IBSBF_2571 (GCA_003590605.1) and O12BS_UW407 (GCA_023076615.1) were excluded due to abnormally long terminal branch lengths and P673 was excluded due to the contradictory phylogenetic position of two assembly versions: the most recent P673 assembly GCA_015698365.1 was assigned to Rso IIB-4NPB clade, while the earlier version GCA_000525615.1 grouped with other Rso IIB-4 *Epipremnum* isolates from the USA.

An additional iteration of clade-specific pangenome analysis was performed for both Rso IIB-4NPB and Rps I-18. Although strain IBSBF1503 (GCA_001587155.1) groups with other Rso IIB-4NPB strains in the RSSC core gene tree, the clade-specific tree revealed this strain is divergent and more properly referred to as an outgroup strain; therefore, this was excluded from a second iteration. Similarly, Rps I-18 strains CIP266_UW505 (GCA_023075875.1) and UW393 (GCA_023076675.1) form a distant outgroup to other Rps I-18 strains; therefore, these were excluded from the second iteration of the Rps I-18 pangenome analysis.

### Mobile Element Prediction in Reference Genome Assemblies

All potential mobile elements, including genes annotated as integrases, transposases, prophage-related genes, and IS elements, were identified and masked for the downstream phylogenetic analysis in the Bakta-annotated genome assemblies for Rps I-18 strain GMI1000 (GCA_000009125.1), Rso IIB-4 strain UW163 (GCA_001587135.1), and Rso IIB-4NPB strain RUN1145. Prophage and IS elements were predicted using the PHASTER, PHASTEST (Zhou et al. 2011; Arndt et al. 2016; Wishart et al. 2023), and ISfinder (Siguier et al. 2006) online tools. All gene annotations containing keywords “phage” OR “transposase” OR “tail” OR “head” OR “integrase” OR “terminase” OR “conjugal” OR “integrase” OR “integrative conjugative element” OR “conjugative transfer” OR “conjugative coupling” were manually searched for and flagged in Geneious Prime (Geneious 2024). The results were converted into BED files, then sorted, merged, and used for masking with Bedtools (Quinlan and Hall 2010). Masked references were used for reference-based contig mapping.

### Phylogenetic Analyses

Reference-based contig mapping was performed for the Rps I-18, Rso IIB-4, and Rso IIB-4NPB clades, as defined by using RSSC and clade-specific core gene trees. Rps I-18 included 100 genomes, with GMI1000 as reference. The Rso IIB-4 clade contained 226 genomes, with UW163 as reference. Rso IIB-4NPB included 179 genomes, with RUN1145 as reference. The Brazilian *Cucumis sativus* isolate IBSBF1503 is referred to as Rso IIB-4NPB (Ailloud et al. 2015) and initially grouped with Rso IIB-4NPB in the species complex tree, and so we initially used this strain as a reference for Rso IIB-4NPB. While more closely related to Rso IIB-4NPB than any other available complete *Rso* genome, IBSBF1503 is clearly an outgroup relative to other Rso IIB-4NPB isolates (Fig. S3). The average pairwise nucleotide diversity is 0.002% between Rso IIB-4NPB genomes, and an order of magnitude greater (0.02%) relative to IBSBF1503. According to progressiveMauve whole-genome alignments, Rso IIB-4NPB isolate RUN1145 differs from RUN1960 and IBSBF1503 by 134 and 1,215 SNPs, respectively. Since IBSBF1503 is too divergent to be considered a member of the same lineage, we reclassified IBSBF1503 as Rso IIB-4. We resequenced five Rso IIB-4NPB strains from Martinique using Nanopore long-read sequencing to generate closed hybrid assemblies and used the complete assembly of Rso IIB-4NPB RUN1145 as a reference for more reliable core genome alignment-based phylogenetic inference of relationships between closely related strains. The Rps I-18 contig mapping using Rps I-18 GMI1000 as reference resulted in 4,760,272 bp consensus alignment with 311 SNPs and 116 parsimony informative sites.

Reads and contigs mappings were performed separately for each assembly with bwa-mem2 (Li 2013; Vasimuddin et al. 2019) or Minimap2 (Li 2016) using asm5 mode, respectively. All resulting BAM alignments were sorted and indexed with Samtools (Li et al. 2009). BCFtools mpileup was used to filter and retain bases with mapping quality above 60 (Li 2011). Variants were called with bcftools-call and filtered by bcftools-filter using the expression TYPE=“snp” && DP>=X && QUAL>30 && AF1>=0.95 with X = 10 for reads and X = 1 for contig mapping. Any alignment column with a gap site was removed. Consensus sequences were obtained using bcftools-consensus, masking the sites with coverage depth below one as identified by Mosdepth (Pedersen and Quinlan 2018). The consensus sequences for all strains in a given clade were concatenated into a FASTA alignment file. Sites containing N characters were trimmed with goalign clean-sites (Lemoine and Gascuel 2021). Recombinant regions were predicted and removed by ClonalFrameML (Didelot and Wilson 2015) using an initial tree calculated by IQtree (Nguyen et al. 2015) under GTR + F + I + R2 substitution model. The final Maximum likelihood trees were calculated from the nonrecombinant alignments for each clade by IQtree using the MFP (ModelFinder Plus) option for the best model search (Kalyaanamoorthy et al. 2017). The contig mapping approach resulted in better resolved and bootstrap supported trees, therefore the trees generated by reads mapping were excluded from further analysis. Tree visualizations were created with ggtree (Yu et al. 2017).

### Tree Dating

Root-to-tip regression and dating of the Rso IIB-4NPB and Rps I-18 nonrecombinant core genome trees were performed with BactDating using 10,000 Markov Chain Monte Carlo iterations (Didelot et al. 2018). TempEst was used to confirm root-to-tip regression results obtained with BactDating, setting the “Best-fitting root” option of TempEst to “R squared”, and for outlier detection by investigating “Residuals” plots (Rambaut et al. 2016). Tree tips with missing sampling date information (Rps I-18 UQRS637_UW745 and UW193, Rso IIB-4NPB RUN0283 and RUN0739) and outliers (Rps I-18 UW393, GMI1000, RUN1974, UW299, RUN1975, RUN1958, RUN1955, RUN0037, RUN0679, and CIP266_UW505; Rso IIB-4NPB RUN0003, RUN0002, RUN2227, RUN2226, RUN1959, RUN1960, RUN0016, RUN2007, RUN2009, RUN2004, RUN2011, RUN0762, RUN1046, RUN1121, RUN0005, RUN1094, RUN1064, RUN1951, RUN1968, RUN1105, RUN1167, and RUN0006) identified by TempEst were dropped from the Rps I-18 and Rso IIB-4NPB alignments and trees were re-calculated. Branches with edge lengths of zero were manually collapsed into a single node by Dendroscope (Huson et al. 2007). A Dates Permutation Test was performed 500 times for each tree with BactDating by randomly generating dates between 2000 and 2012 and randomly assigning these to tree tips while keeping the tree root (option: updateRoot = F). The simulated trees were compared to the actual dating model using the modelcompare function of BactDating. Divergence times of select nodes in Fig. 2 were visualized in matplotlib using plt.errorbar function.

### CRISPR-Cas System Identification

CRISPR-Cas systems were identified with Bakta annotation of Rso IIB-4 and Rso IIB-4NPB genomes. CRISPR repeats and spacers were then annotated in two closed references Rso IIB-4-UW163 and Rso IIB-4NPB-RUN1145 using the CRISPRloci workflow (Alkhnbashi et al. 2021). The spacer sequences identified with CRISPRloci were then used for BLASTn searches against the RefSeq Phage database downloaded from PhageScope (Wang et al. 2024a), NCBI Nucleotide collection (nr_nt), and Environmental samples (env_nt) databases accessed in June 2024. The extracted spacers were also used for INPHARED database (Cook et al. 2021) searches using the SpacePHARER (Zhang et al. 2021) toolkit in August 2024.

### Inter and Intraspecific Recombination Prediction

Potential homologous recombination events within and between Rso IIB-4 and Rps I-18 lineages were investigated using the RECOPHY toolkit (Sakoparnig et al. 2021). Two datasets were analyzed: Rso IIB-4 alone and in combination with Rps I-18, using Rso IIB-4NPB-RUN1145 as a reference in each case. Prior to RECOPHY execution, all genome pairs with a Mash distance (Ondov et al. 2016) below zero were identified and one member of each pair was then randomly removed from the dataset. The final datasets included 49 genomes for the Rso IIB-4 analysis alone, and with an additional 64 Rps I-18 genomes for the merged analysis. The regions of core alignment with increased and reduced density of SNPs per 1 kb block were extracted and investigated separately. In order to find discrepancies with the core phylogeny, a simple neighbor-joining tree was calculated for each of these regions using Geneious Prime software (Geneious 2024).

### Pangenome-Wide Association Testing

Scoary and Pyseer pan-GWAS analyses were used to investigate associations between gene presence and absence patterns and the host or location of strain isolation (Brynildsrud et al. 2016; Lees et al. 2018). The broad (country) and fine-scale (village) locations and all taxonomic levels of the host of isolation or environmental substrate were converted from the metadata table into a binary traits table using the pandas.get_dummies function. For example, a strain isolated from a tomato plant in China would receive the value “1” in the columns China, *Eudicots*, *Asterids*, S*olanales*, *Solanaceae*, *Solanum*, and *S. lycopersicum*, and receive the value “0” in all other columns of the traits table. This traits table was used as input for both Scoary and Pyseer pan-GWAS analyses, along with the gene presence and absence table from Panaroo.

The significance of candidates identified with Scoary was assessed after multiple test corrections: individual (naive) *P*-value, Bonferroni adjusted *P*-value, Benjamini–Hochberg adjusted *P*-value, entire range of pairwise comparison *P*-values, and empirical *P*-value from 100 permutations, using thresholds of *P* = 0.05 for all corrections. Significant genes identified by Scoary after all the corrections were further filtered using 95% sensitivity and 70% specificity thresholds. A lower specificity threshold was chosen due to the nature of the trait dataset: the traits table simply reflects the known host of isolation and the location of isolation. The actual host range of a particular strain may include additional hosts, closely or distantly related, just as closely related strains may be present in vastly different geographic regions. Since the Scoary and Pyseer pan-GWAS analyses aim to identify genes linked with survival in a particular host, environment, or substrate, the sensitivity should be high. Those same genes may also be present in strains isolated from other plants; however, therefore specificity is reduced. For example, a gene whose product is strongly associated with tomato (*S. lycopersicum*) infection should be overrepresented among tomato isolates in the dataset. Therefore we seek genes present in 95% of tomato isolates. We reduced the specificity threshold to 70% to allow for instances where another strain carries that same gene and is also capable of infecting tomato, but happens to have been isolated from a different host.

For the Pyseer analysis, the distance matrix from the RSSC core gene tree and the number of patterns for the likelihood ratio test (LRT) were calculated using phylogeny_distance.py and count_patterns.py scripts downloaded from the Pyseer GitHub page, accessed on January 2024 (Lees et al. 2018). The pattern count resulted in 6,075 patterns for the RSSC, leading to the likelihood ratio test (LRT) *P*-value threshold of 3.8e-08 = 0.05/(patterns_number*traits_number). Hits with effect size (beta value) under 0.2 and phenotypic variance values (variant_h2) above 0.5 were filtered out.

Pan-GWAS of gene presence/absence data is an effective tool for the identification of genes associated with traits of interest but is limited to the accessory genome and does not test for trait associations within the core genome and intergenic regions. This gap is filled with a kmer-based association test. Analogously to the method used for gene presence/absence data, Pyseer was applied to identify the kmers significantly associated with any host or location of isolation across the RSSC, using the same binary metadata table as described for the pan-GWAS analysis. The initial set of 31 bp long kmers was generated for all the RSSC genomes using unitig-caller ^1^. The distance matrix from the species complex core genes tree and the number of patterns for the LRT were calculated using Pyseer phylogeny_distance.py and count_patterns.py scripts (Lees et al. 2018). The pattern count reported 1,484,018 patterns for the RSSC kmers, producing a LRT *P*-value threshold of 5.0e-10 = 0.05/(patterns_number*traits_number). Hits with effect size (beta value) under 0.2 and phenotypic variance values (variant_h2) above 0.5 were filtered out. The presence and positions of significant kmers in Rso IIB-4NPB reference strain RUN1145, Rso IIB-4 reference strain UW163, and Rps I-18 reference strain GMI1000 were identified and stored in BED files using a custom Python script. For visualization purposes, all kmers mapped on a genome within a 1 kb distance were merged using the bedtools-merge function (Quinlan and Hall 2010).

The pan-GWAS and kmer-based tests identify many genes associated with isolation from specific hosts. These tests vary in how they control for population structure, but some genes were predicted to have associations with particular traits using multiple methods. Separate association tests were performed using either spatial location or host/substrate of isolation metadata. We noticed that many genes have overlapping predictions using both metadata types, particularly where it concerns isolates recovered from soil or water. We also found overlapping host associations at multiple taxonomic levels; for example, gene presence associated with *S. lycopersicum* isolation often overlaps with gene presence associated with *Solanaceae* isolation. All overlaps were investigated using a custom Python script and visualized in an Upset plot using the UpSetR R package (Conway et al. 2017). For the genes assigned to multiple nested traits, the lowest level of taxonomic identification (eg species rather than genus) or the finest level of geographical scale (eg village rather than country) for which significance was identified were chosen. Overlaps within the Scoary, Pyseer pan-GWAS, and kmer-based outputs were identified using the same strategy.

### Identification of ICEs

Complete ICEs were identified in complete and draft RSSC genomes using the following criteria: the presence of a Type 4 secretion system (T4SS) required for conjugal transfer between strains, a *mob* family relaxase and integrase, and the presence of 8 bp direct repeats (TTTTTTAT) encompassing the predicted ICE (Colombi et al. 2021). The flanking repeat (TTTTTTAT) was identified using a custom python script and is described as an ICE insertion site for Rps I-18 GMI1000 in the ICEberg 3.0 database (Wang et al. 2024b). We note this repeat occurs between 50 and 70 times elsewhere in each RSSC genome, though this does not preclude them from being integration sites. Some ICE integrases are known to be promiscuous and may target different repeats as attachment sites across the genome (eg ICE Tn916) (Lu and Churchward 1995). In genomes where the repeat was not identified within 10 kb of the T4SS, relaxase, and integrase, putative insertion sites were manually identified. MOBscan was used for the identification of relaxases (Garcillán-Barcia et al. 2020), followed by BLASTn sequence homology searches for a T4SS and adjacent integrases (Altschul et al. 1997) using Rso IIB-4NPB RUN1960 T4SS and integrase sequences as BLASTn queries, followed by T4SS locus confirmation with the TXSScan module of the MacSyFinder toolkit (Abby et al. 2016). The genomic context of putative ICEs in the RSSC assemblies was inferred by aligning 50 kb upstream and downstream flanking regions surrounding putative ICEs in Rps I-18 GMI1000, Rso IIB-4NPB RUN1145, and Rso IIB-4NPB RUN1960 to the genomes. The locus structure of putative ICEs was visualized with the ggtree::geom_motif R function (Yu et al. 2017). DIAMOND alignments of the Rps GMI1000 ICE *tra*, relaxase, and integrase genes were used for ICE identification in the draft assemblies. A set of 18 ICEs disrupted by contig breaks in draft assemblies were excluded from the analysis but are listed in Table S14. The set of 264 ICEs identified in all draft assemblies were initially aligned by FFT-NS-1. Seventeen nonredundant representative ICEs in Caribbean and South American genomes were aligned and visualized by MUMmer using pgv-mummer in pyGenomeViz. The involvement of ICE cargo genes in specific metabolic pathways was predicted using KEGG Orthology assignment with protein sequences from the cargo regions of interest, performing searches in the database for the genus *Ralstonia* (taxa ID: 48736).

### Geographical Metadata Display

Information about the geographical region of isolation for each strain was obtained from the country of isolation metadata collected for the RSSC strains set as described above. Counties of isolation were converted into continents using pycountry Python package (Theune et al.). The “Caribbean” region of isolation was manually assigned for all Martinique isolates to highlight the region of interest.

Longitude and latitude of the sampling site were recorded for 83/500 strains sampled in Martinique and French Guiana. For the remaining 417/500 strains, the commune (administrative district) or the nearest village was listed in the strain metadata. For these cases, approximate longitude and latitude values were retrieved using the geolocator function of the geopy.geocoders Python package using the commune or nearest village names (Esmukov). Commune maps of Martinique and French Guiana in geojson format were downloaded from the France Geojson collection (Butler et al.). The actual and estimated longitude and latitude values for each isolate were visualized with the geopandas package (Joris Van den Bossche et al. 2024).

## Supplementary Material

evag192_Supplementary_Data

## Data Availability

Sequence data generated for this work are available via NCBI SRA Accession PRJNA1196686. Annotated assemblies are available via Zenodo at https://zenodo.org/records/15619409.
